# Exploring Beneficial Properties of the Bacteriocinogenic *Enterococcus faecium* ST10Bz Strain Isolated from *Boza*, a Bulgarian Cereal-Based Beverage

**DOI:** 10.3390/microorganisms8101474

**Published:** 2020-09-25

**Authors:** Samantha Joy D. Valledor, Jorge Enrique Vazquez Bucheli, Wilhelm H. Holzapfel, Svetoslav Dimitrov Todorov

**Affiliations:** ProBacLab, Advanced Green Energy and Environment Institute (AGEE), Handong Global University, Pohang, Gyeongbuk 791-708, Korea; sdvalledor@up.edu.ph (S.J.D.V.); jorge_jbv@hotmail.com (J.E.V.B.); wilhelm@woodapple.net (W.H.H.)

**Keywords:** bacteriocins, anti-*Listeria* activity, beneficial properties

## Abstract

The bacteriocin-producing strain *Enterococcus faecium* ST10Bz, isolated from *boza*, a Bulgarian cereal-based beverage, exhibited strong activity against *Listeria* strains, vancomycin-resistant and other *Enterococcus* strains, but not against most of the other lactic acid bacteria (LAB) strains included in the test panel. Bacteriocin ST10Bz was proven as a stable antimicrobial, even after exposure to various environmental conditions, including varying pH values, temperatures, and commonly used chemicals in industry and laboratory practice. Bacteriocin activity against *L. monocytogenes* ATCC^®^15313™ was recorded at 25,600 AU/mL when the producer strain was cultured in MRS broth at 25 °C and 30 °C, and 19,200 AU/mL, when cultured at 37 °C. Additionally, bacteriocin ST10Bz exhibited bactericidal mode of action when added to actively growing cultures of *L. monocytogenes* ATCC^®^15313™ and *Enterococcus faecalis* 200A. *E. faecium* ST10Bz was susceptible to the antibiotics kanamycin, gentamycin, ampicillin, streptomycin, tylosin, chloramphenicol, clindamycin, tetracycline, and vancomycin; with no evidence for *van*A, B, C, D, E, or G genes. PCR analysis of DNA from strain ST10Bz generated positive results for presence of some bacterial adhesion genes, including *map*, *mub* and *ef-tu*, as well as the gamma aminobutyric acid (GABA) production-related gene, *gad*. Under simulated gastrointestinal conditions in single and co-culture with *L. monocytogenes* ATCC^®^15313™ and *E. faecalis* 200A, *E. faecium* ST10Bz showed a high survival rate and the ability to reduce the viable numbers of the two test strains.

## 1. Introduction

Several species belonging to the group of Lactic acid bacteria (LAB) are Generally Recognized As Safe (GRAS) [1], having different biotechnological applications such as in the food and feed fermentation processes, and can produce metabolites of commercial importance, including organic acids, antimicrobial and aromatic compounds, exopolysaccharides, vitamins, and bio-active peptides, that may contribute to the sensory, nutritional and safety properties of fermented food products [2]. Moreover, lactic acid, hydrogen peroxide, diacetyl, and bacteriocins are the most studied antimicrobials produced by different LAB strains [3]. Microbial metabolites, including the bioactive compounds produced during fermentation processes, as well as cell structures, are collectively termed as postbiotics [4]. These comprise all the non-viable structures and components of microbial cells. Antibiotics, described as of low-molecular weight and mostly non-proteinaceous, are among the most popular antimicrobial metabolites produced by microorganisms. These antimicrobial compounds were even used as growth promoters in animal farming, However, were eventually banned in several countries, as these were considered to selectively promote antibiotic resistance of bacterial populations [5]. According to the World Health Organization (WHO), the emergence and spread of resistance has worsened by allowing antibiotics to be bought over the counter [6]. Moreover, the lack of urgency in addressing this problem may lead to a post-antibiotic era where common microbial infections may become highly problematic, probably like the time before the discovery of antibiotics.

Taking into consideration the increasing concerns related to antibiotic resistance, the search for alternatives to antibiotics has become a prioritized trend in the scientific community. Antimicrobial peptides produced by different bacterial cultures, known as bacteriocins, became popular even before the term postbiotics was coined [7]. These antimicrobial peptides are characterized by either broad or narrow spectra of activity, making the exploration of this field considerable as the next frontier in combating pathogens [8]. Generally, bacteriocins are biologically active, small, ribosomally-synthesized polypeptides with a bactericidal mode of action and are mostly active against species closely related to the producer strain [9,10]. Aside from being potential alternatives to antibiotics, bacteriocins are already used as bio-preservative agents in food and feed industries, being significantly potent against other detrimental bacteria, including various pathogenic and even some antibiotic-resistant strains [10]. In most cases, the antimicrobial modes of action of bacteriocins are related to specific cell surface receptor recognition, and thus resistance to these polypeptides is not easily generated when compared with antibiotics [11].

In the medical field, listeriosis is a relatively rare but highly dangerous food-borne infection caused by *Listeria monocytogenes* [12]. Different countries, specifically in the European Union and the USA, maintain zero-tolerance for *L. monocytogenes* in ready-to-eat food, especially on dairy products [13]. Infection with *L. monocytogenes* is considered high risk for specific population groups, in particular for pregnant women, elderly, and immunocompromised individuals. Such infections may be established in the form of neuro-meningeal (meningitis or encephalitis) and maternal-neonatal (intrauterine infection, or spontaneous abortion) conditions; this could also include febrile gastroenteritis and may even lead to brain infection, sepsis, and death [9]. Moreover, pets may also harbor, get infected, and spread listeriosis [14,15].

On the other hand, several species in the genus *Enterococcus*, considered as commensals in the gastrointestinal tracts of animals and humans, are widely distributed in foods, soil, water, and plants [16], while some strains have been commercialized as safe probiotics [17]. However, some *Enterococcus* strains (mainly belonging to the species *E. faecalis* and *E. faecium*) have also been recognized as clinically significant pathogens in human and veterinary medicine. In addition, nosocomial enterococcal infections and the emergence of the multi-drug resistant strains, such as vancomycin-resistant enterococci (VREs), have become a rapidly emerging health issue. Specifically, VREs may obtain selective advantage over other organisms in an antibiotic-exposed environment, and eventually may transfer virulence genes to other microorganisms, particularly to pathogenic microorganisms [18].

Emergence of listerial and enterococcal infections, along with the incapacity of antibiotics to combat these infections, has prompted the scientific community to find alternative ways to combat these and related infections. Utilization of beneficial microorganisms and their metabolites against these pathogens is considered as a viable alternative for potential therapeutic treatment of these infections. In this study, isolation of antimicrobial metabolite-producing bacteria with strong activity against strains of *Listeria* and *Enterococcus*, including characterization of the produced antimicrobial/s and investigations on the potential safety and other beneficial properties of the isolated strains were performed.

## 2. Materials and Methods

### 2.1. Isolation of Bacteriocinogenic Strains and their Inhibitory Activity

Isolation of antimicrobial-producing LAB from the Bulgarian cereal-based beverage, *boza*, was performed by a triple layer method according to dos Santos et al. [19]. Product samples were obtained from a medium scale producer in the region of Belogradchik, in the North-West of Bulgaria. *Boza* samples were serially diluted on 1X PBS (Lonza, Basel, Switzerland). Dilutions were spread plated on de Man, Rogosa and Sharpe (MRS) agar (Becton, Dickinson and Company—BD, Franklin Lakes, NJ, USA), overlaid with 1% agar (m/v) (LPS Solution, Daejeon, South Korea), and incubated aerobically at 37 °C for 48 h. Plates containing distinguishable colonies were chosen and covered with Brain Heart Infusion (BHI) (BD) supplemented with 1% agar, inoculated with the test strains, *L. monocytogenes* ATCC^®^15313™ and *E. faecalis* 200A (HEM Inc., Pohang, South Korea), respectively, to a final concentration of 10^5^ CFU/mL, and incubated aerobically at 37 °C for 24 h. Colonies with inhibition zones against the test strains were selected and sub-cultured in MRS broth at 37 °C for 24 h.

Based on the description of dos Santos et al. [19], the isolated strains were evaluated based on their ability to produce bacteriocins against the mentioned two test strains (*L. monocytogenes* ATCC^®^15313™ and *E. faecalis* 200A). Briefly, cell free supernatants (CFS) from overnight cultures of the selected isolates were obtained after centrifugation (10,000× *g*, 5 min, 20 °C). The CFSs were heat treated at 80 °C for 10 min to inactivate the possibly produced proteolytic enzymes and hydrogen peroxide. The heat-treated CFSs were tested against *L. monocytogenes* ATCC^®^15313™ and *E. faecalis* 200A by the agar spot test. All isolates of interest were evaluated, based on standard morphological, biochemical, and physiological tests as recommended in Bergey’s Manual [20], including Gram-staining, catalase test and production of CO_2_ from glucose.

Confirmation of the bacteriocinogenic nature of isolated strains was done as described by dos Santos et al. [19] with modifications. Heat-treated CFS at 80 °C for 10 min was prepared as described. Resulting CFS’s were filtrated using Minisart^®^ 0.22 µm syringe filter (Sartorius AG, Goettingen, Germany). The bacteriocin activity titer was determined by serial two-fold dilutions of treated CFS with 0.1 M phosphate buffer (KH_2_PO_4_/K_2_HPO_4_; pH 6.5), evaluated against *L. monocytogenes* ATCC^®^15313™ and *E. faecalis* 200A by the agar spot test. The active units of bacteriocins were expressed as arbitrary units per milliliter (AU/mL), calculated as: AU/mL = (D^n^ × 1000)/p, where D is the type of dilution (two-fold), n is the first dilution with no inhibition zone, p is the volume of the CFS spotted in μL, and 1000 is the conversion factor between μL and mL.

The proteinaceous nature of the investigated antimicrobial/s was confirmed as described by dos Santos et al. [19] with addition of proteolytic enzymes comprising 100 μL/mL of Proteinase K (Qiagen, Hilden, Germany), and 0.1 mg/mL of pepsin and α-chymotrypsin, respectively (Sigma-Aldrich Co., St. Louis, MO, USA) to the CFS. Incubation was at 37 °C for 1 h followed by heat treatment at 98 °C for 5 min to denature the added proteolytic enzymes. Antimicrobial activity was determined against *L. monocytogenes* ATCC^®^15313™ and *E. faecalis* 200A as described above. Original CFS, not treated with the enzymes, served as control.

### 2.2. Selection and Identification of Bacterial Isolates

Bacterial isolates exhibiting bacteriocinogenic potential were grown in MRS broth at 37 °C for 24 h. The total DNA was extracted using ZR Fungal/Bacterial DNA Kit (Zymo Research, Irvine, CA, USA) according to the manufacturer’s manual. Concentrations of the extracted DNA were determined using nanodrop (Spectrostarnano, Ortenberg, Germany) and were subsequentially subjected to DNA fingerprinting by rep-PCR and RAPD-PCR on a Veriti™ 96-well thermal cycler (Thermo Scientific, Waltham, MA, USA). Differentiation between the studied isolates was performed by rep-PCR (5′-(GTG)_5_ -3′) and RAPD-PCR (OPL-01: 5′-GGC ATG ACC T-3′; OPL-03: 5′-CCA GCA GCT T-3′; OPL-09: 5′-TGC GAG AGT C-3′; OPL-11: 5′-ACG ATG AGC C-3′) (all primers obtained from Macrogen Co., Ltd., Daejeon, Korea) following the conditions recommended by de Moraes et al. [21]. The obtained amplicons were separated via gel electrophoresis (GH-200 Genera Biosystems, Victoria, Australia; Elite 300 Plus Power Supply, Wealtec Bioscience Co., Ltd., Taiwan) and visualized using Omega Lum™ G gel documenter (Aplegen, Inc., San Francisco, CA, USA). The strain selected for future study was identified using 16S rRNA sequencing performed by Solgent Co., Ltd. (Daejeon, South Korea) as a commercial service.

### 2.3. Detection of Known Bacteriocin Genes in the Genome of the Studied Strain

The selected strain was investigated for the presence of already known bacteriocin genes using the primers related to enterocin A (EntA/F: 5′-GAG ATT TAT CTC CAT AAT CT-3′; EntA/R: 5′-GTA CCA CTC ATA GTG GAA-3′), Enterocin B (EntB/F: 5′-GAA AAT GAT CAC AGA ATG CCT A-3′; EntB/R: 5′-GTT GCA TTT AGA GTA TAC ATT TG-3′), enterocin P (EntP/F: 5′-ATG AGA AAA TTA TTT AGT TT-3′; EntP/R: 5′-TTA ATG TCC CAT ACC TGC CAA ACC-3′), enterocin L50B (EntL50B/F: 5′-ATG GGA GCA ATC GCA AAA TTA-3′; EntL50B/R: 5′-TAG CCA TTT TTC AAT TTG ATC-3′), nisin Q (nis/F: 5′-ATG AGT ACA AAA GAT TTCAAC TT-3′; nis/R: 5′-TTA TTT GCT TAC GTG AAC GC-3′) and pediocin PA1 (Pedpro/F: 5′-CAA GAT CGT TAA CCA GTT T-3′; Ped1041/R: 5′-CCG TTG TTC CCA TAG TCT AA-3′) according to Aymerich et al. [22], Du Toit et al. [23], Gutierrez et al. [24] and Todorov et al. [25]. Reactions were performed, profiled, and visualized as described above [23,24]. For verification of the identity of amplified genes, the generated amplicons were purified using QIAquick^®^ PCR purification kit (Qiagen) and were submitted for sequencing to Macrogen Co., Ltd., as a commercial service. The sequences were compared and identified using BLAST (GenBank, National Center for Biotechnology Information, Bethesda, MD, USA).

### 2.4. Evaluation of Bacteriocin Production

#### 2.4.1. Growth Production Experiment

The dynamics of bacterial growth, bacteriocin production and acidification of the cultural environment were observed by growing the strain at 37 °C in MRS broth for 24 h. Bacterial growth was determined by changes in the Optical Density (OD) measured at 600 nm in a spectrophotometer (Spectrostarnano), simultaneously with recording changes in the pH every hour using pHspear (Oakton Instruments, IL, USA), and bacteriocin production was evaluated against *L. monocytogenes* ATCC^®^15313™ and *E. faecalis* 200A every 3 h by titration, as mentioned above.

#### 2.4.2. Stability of Antimicrobial Peptides

CFSs were exposed to a variety of environmental conditions, including different temperatures, pH values, and presence of selected chemicals, in order to evaluate the stability of the antimicrobial/s present. The methods described by dos Santos et al. [19] were applied. For the evaluation of the effect of temperature on the stability of antimicrobial peptide/s produced by the studied strain, its CFS was incubated at 5.4, 30, 37, 80, and 100 °C, for 1 and 2 h, as well as with autoclaving at 121 °C for 15 min. In a separate experiment, the effect of different pH-values was determined following adjustment of the CFS to pH 2.0, 4.0, 6.0, 8.0, and 10.0 (with 1M NaOH or 1M HCl). Lastly, for evaluation of the effect of selected chemical exposure on the stability of antimicrobial/s, CFS was respectively supplemented with 10 mg/mL sodium dodecyl sulfate (SDS) (Junsei Chemical Co., Ltd., Tokyo, Japan), sodium chloride (NaCl) (Daejung Chemicals, Siheung, South Korea), Tween 20 (Daejung), Tween 80 (Duksan Company, Gyeonggi, South Korea), skim milk (BD), and glycerol (Junsei). All the preparations for pH and chemical experiments were incubated at 37 °C for 1 h. After incubation, pH-values were corrected back to neutral (5.5–6.5), and the total antimicrobial activity evaluated using *L. monocytogenes* ATCC^®^15313™ and *E. faecalis* 200A as indicator strains as described above. Original, non-treated CFS served as control.

In addition to the previously described characteristics of the bacteriocin/s, the stability of the antimicrobial protein/s stored at −20 °C was evaluated over time. Briefly, CFS of *E. faecium* ST10Bz was obtained as described above and stored at −20 °C. After preparation, its antimicrobial activity against *L. monocytogenes* ATCC^®^15313™ was periodically evaluated over 11 months as described.

#### 2.4.3. Optimization of Culture Condition for Bacteriocin Production

Bacteriocin production is dependent on the conditions of the culture medium. The optimal commercial medium for bacteriocin production was determined by growing the strain on different culture media, including MRS, BHI, BL (MBcell), Luria-Bertani (LB) (BD), M17 (BD) with 0.5% glucose, and 5% and 10% Skim Milk (BD) for 24 h at 37 °C. Subsequently, optimal production temperature, pH, and medium composition were evaluated. For the optimal incubation temperature, the bacteriocinogenic strain was grown in MRS broth at 25 °C, 30 °C, and 37 °C. For optimal pH of the culture medium, the strain was grown in MRS broth adjusted to pH 2.0, 4.0, 6.0, 8.0, and 10.0 respectively (using either 1M NaOH or 1M HCl). For the optimal medium composition, the strain was grown in 19 differently designed variations of MRS broth, as listed in Table S1. All the cultures were incubated at 37 °C for 18 h, except for the setups for optimal incubation temperature. CFSs were prepared as described above, with the exception of skim milk cultures, centrifuged for 15 min. The varying pH values of the culture supernatants were adjusted to neutral (5.5–6.5). Lastly, all the CFSs were heat treated at 80 °C for 10 min. The activity of the produced bacteriocin against *L. monocytogenes* ATCC^®^15313™ was determined and expressed as AU/mL as described above.

#### 2.4.4. Bacteriocin Mode of Action

The effect of the studied bacteriocin/s on the actively growing cultures of *L. monocytogenes* ATCC^®^15313™ and *E. faecalis* 200A was determined according to Favaro et al. [26]. For the purpose of the experiment, 50 mL BHI broth was inoculated with 1% (v/v) cultures of *L. monocytogenes* ATCC^®^15313™ or *E. faecalis* 200A and incubated at 37 °C. Changes in turbidity were monitored by spectrophotometer (Spectrostarnano) at OD600 nm and recorded every hour for 12 h. 20% filter-sterilized (0.22 μm, Sartorius AG) CFS was added to the cultures of *E. faecalis* 200A and *L. monocytogenes* ATCC^®^15313™ in the beginning of the exponential growing phase, specifically after 3 and 5 h from inoculation, respectively. Experimental controls represented the growth of *L. monocytogenes* ATCC^®^15313™ and *E. faecalis* 200A without the addition of CFS of the bacteriocinogenic strain.

#### 2.4.5. Adsorption of Bacteriocin to *Listeria monocytogenes* ATCC^®^15313™

The initiation of interaction between bacteriocin and target cells was assessed as described by Todorov et al. [27]. Adsorption of bacteriocin to the test organisms was observed with varying external factors, including temperature, pH and presence of selected chemicals. Cells from an 18 h culture of *L. monocytogenes* ATCC^®^15313™ were obtained by centrifugation (10,000× *g*, 10 min, 20 °C) and were resuspended with the original volume in 0.1 M phosphate buffer (KH_2_PO_4_/K_2_HPO_4_; pH 6.5) for varying temperature and chemical tests, and in saline solution (0.85%, w/v NaCl) for the varying pH test. CFS of the evaluated bacteriocinogenic strain (6400 AU/mL) and suspension of *L. monocytogenes* ATCC^®^15313™ were mixed at a 1:1 proportion. For the evaluation of the effect of temperature on the adsorption of bacteriocin to the surface of *L. monocytogenes* ATCC^®^15313™, mixtures (*L. monocytogenes* ATCC^®^ 15313™ cell suspension with CFS) were incubated at 5.4, 27, 30, 37, 40, 50, 60, 70, 80, 90, and 100 °C, respectively. For testing the effect of pH, mixtures were adjusted to pH values of 2.0, 4.0, 6.0, 8.0, and 10.0. For the evaluation of the effect of different chemicals, 1% (w/v) of NaCl, K_2_HPO_4_, KH_2_PO_4_ (Daejung), MgCl_2_ (Junsei), SDS, β-mercaptoethanol (Sigma-Aldrich), and 80% ethanol (Merck, Kenilworth, NJ, USA) were added to the mixtures. All preparations for experiments on the effect of pH and chemicals were incubated at 37 °C for 1 h. After exposure to different conditions, CFS was obtained by centrifugation (100,000× *g*, 5 min, 20°C), and the obtained supernatants were corrected to neutral pH (5.5–6.5; for the pH experimental setups), and all heat treated at 80 °C for 10 min. Activity of the remaining bacteriocin in the supernatant was evaluated in a similar manner, as described above. The percentage adsorption of bacteriocin to *L. monocytogenes* ATCC^®^15313™ was calculated using the formula: % adsorption = 100 – ((bacteriocin activity after treatment in AU/mL/bacteriocin activity before treatment in AU/mL) × 100).

#### 2.4.6. Spectrum of Activity

In addition to *L. monocytogenes* ATCC^®^15313 and *E. faecalis* 200A, different strains from the culture collection of Handong Global University (Pohang, South Korea), HEM Inc., KCTC (Jeongeup, South Korea), KACC (Jeollabuk-do, South Korea) and ATCC (Manassas, VA, USA) were used as indicators for evaluation of bacteriocin activity. Growth origin and growth conditions for evaluated microorganisms in the determination of spectrum of activity are specified in Table S2. Evaluation of the activity of the bacteriocin against the mentioned pool of strains was performed as described above.

### 2.5. Safety Tests and Detection of Additional Beneficial Properties

#### 2.5.1. Antibiotic Susceptibility Test

The antibiotic susceptibility test for *E. faecium* ST10Bz was performed using the agar diffusion method according to the guidelines of the Clinical and Laboratory Standards Institute (CLSI; www.clsi.org) as suggested by Arellano et al. [28]. Two-fold serial dilutions of the antibiotics ampicillin, gentamycin, kanamycin, vancomycin, erythromycin, tetracycline, chloramphenicol, clindamycin, streptomycin, and tylosin were mixed on Mueller-Hinton Agar (MBcell). Ten μL of the cell suspensions from the overnight cultures of *E. faecium* ST10Bz and a quality control (QC) strain, *E. faecalis* ATCC^®^29212™ (approximately 10^7^ CFU/mL), were spot plated on the antibiotic test plates and incubated at 37 °C for 24 h. The minimum inhibitory concentration (MIC) of each antibiotic was compared to the European Food Safety Authority (EFSA) breakpoint for *E. faecium* [29], while ranges of MIC for the QC strain, *E. faecalis* ATCC^®^29212™, were based on the CLSI manual [30] as listed in Table 1.

As additional antibiotic safety assessment the evaluated strain was tested for the presence of vancomycin resistance genes: vanA (VanAB/F: 5′-GTA GGC TGC GAT ATT CAA AGC-3′; VanA/R: 5′-CGA TTC AAT TGC GTA GTC CAA-3′), van B (VanAB/F: 5′-GTA GGC TGC GAT ATT CAA AGC-3′; VanB/R: 5′-GCC GAC AAT CAA ATC CTC-3′) [31], van C (VanC/F: 5′ – ATC CAA GCT ATT GAC CCG CT-3′; VanC/R: 5′-TGT GGC AGG ATC GTT TTC AT-3′) [32], van D (VanD/F: 5′-TGT GGG ATG CGA TAT TCA A-3′; VanD/R: 5′-TGC AGC CAA GTA TCC GGT AA-3′) [33], van E (VanE/F: 5′-TGT GGT ATC GGA GCT GCA G-3′; VanE/R: 5′-GTC GAT TCT CGC TAA TCC-3′) [34], and van G (VanG/F: 5′-GAA GAT GGT ACT TTG CAG GGC A-3′; VanG/R: 5′-AGC CGC TTC TTG TAT CCG TTT T-3′) [35]. Reactions were performed, profiled, and visualized as described above [31,32,33,34,35]. For verification of the identity of amplified genes, the generated amplicons were purified using QIAquick ^®^ PCR purification kit (Qiagen) and submitted for sequencing to Macrogen Co., Ltd. as a commercial service. The sequences were compared and identified using BLAST (GenBank).

#### 2.5.2. Hemolysis Test

The ability of the bacteriocinogenic strain *E. faecium* ST10Bz to lyse blood was assessed as described by Arellano et al. [28]. Briefly, overnight cultures of *E. faecium* ST10Bz and *Bacillus cereus* ATCC^®^27348™ (positive control for β-hemolysis) were streak plated on agar supplemented with 5% defibrinated sheep blood (Synergy Innovation Co. Ltd., Seongnam-si, South Korea). After incubation at 37 °C for 24 h, the hemolytic activity of each isolate was assessed and classified as total or β-hemolysis (clear halos around the colonies), partial or α-hemolysis (greenish halos around the colonies), or γ-hemolysis (absence of hemolysis) [28].

#### 2.5.3. Biogenic Amine Production Test

Evaluation of *E. faecium* ST10Bz for production of biogenic amines (BA) was assayed as described by Arellano et al. [28]. *E. faecium* ST10Bz and *Escherichia coli* ATCC^®^25922™ (positive control) were streaked onto different media containing 1% of the amino acid precursors of histamine, tyramine, cadaverine, and putrescine, namely L-histidine (Daejung Chemicals), L-tyrosine (Samchun Chemicals, Seoul, South Korea), L-lysine (Samchun Chemicals), and L-ornithine (Sigma-Aldrich), respectively. The plates were incubated at 37 °C for 24 h. Purple discoloration of the media, primarily due to the increase in pH as decarboxylation of the amino acids occurs, was considered as qualitative evidence of BA production.

#### 2.5.4. Detection of Adhesion and Gamma Aminobutyric Acid (GABA) Production Related Genes

The primers targeting amplification of genes MapA (Map/F: 5′-TGG ATT CTG CTT GAG GTA AG-3′; Map/R: 5′-GAC TAG TAA CGC GAC CG-3′) and Mub (Mub/F: 5′-GTA GTT ACT CAG TGA CGA TCA ATG-3′; Mub/R: 5′-TAA TTG TAA AGG TAT AAT CGG AGG-3′) (mucus adhesion genes), and EF-Tu (EfTu/F: 5′-TTC TGG TCG TAT CGA TCG TG-3′; EfTu/R: 5′-CCA CGT AAT AAC GCA CCA AC-3′) (adhesion-like factor), and prgB (prgB/F: 5′-GCC GTC GAC TCG AGG AGA ATG ATA CAT GAA T-3′; prgB/R: 5′-CCT GCG GCC GCG TCC TTC TTT TCG TCT TCA A-3′) (aggregation substance gene), EF2662 (EF2662/F: 5′-GGC GTC GAC CAC TTA AAC TGA TAG AGA GGA AT-3′; EF2662/R: 5′-CGC GCC GCA ATT AAT TAT TAA CTA GTT TCC-3′) (choline-binding protein gene), EF1249 (EF1249/F: 5′-GCG GTC GAC AAA CGA GGG ATT TAT G-3′; EF1249/R: 5′-CTG GCG GCC GCG TTT AAT ACA ATT AGG AAG CAG A-3′) (fibronectin-binding protein) and EF2380 (EF2380/F: 5′-GCG GTC GAC ATC TAT GAA AAC AAT-3′; EF2380/R: 5′-TCC GCG CCG CCT TAA ACT TTC TCC TT-3′) (membrane-associated zinc metalloprotease gene) were used to determine the presence of the mentioned genes on the genome of the selected strain. DNA from the strain was obtained and quantified as described above. PCR reactions were performed, profiled, and analyzed in the same manner as above, with PCR conditions following the recommendations of Todorov and Dicks [36] and de Castilho et al. [37].

The presence of genes related to the production of gamma aminobutyric acid (GABA), a neurotransmitter inhibitor claimed to have an immunomodulatory effect on the body, was evaluated according to Bajić et al. [38]. Briefly, amplification of the genes for glutamate decarboxylase (gad) production, an enzyme used to convert glutamate to GABA, was performed (using the primers CoreF: 5-CCT CGA GAA GCC GAT CGC TTA GTT CG-3′ and CoreR: 5′-TCA TAT TGA CCG GTA TAA GTG ATG CCC-3′). PCR reactions were profiled and analyzed in the same manner as described earlier [38].

#### 2.5.5. Gastrointestinal Simulation

The survivability of the bacteriocinogenic *E. faecium* ST10Bz, *E. faecalis* 200A and *L. monocytogenes* ATCC^®^15313™ under the simulated gastrointestinal tract (GIT) conditions was determined by adaptation of the methods of dos Santos et al. [19]. *E. faecium* ST10Bz, *E. faecalis* 200A and *L. monocytogenes* ATCC^®^15313™ were cultured overnight in an appropriate culture liquid medium. CFU/mL was determined for all studied strains in order to determine the initial viable cell counts by plating serial dilutions on MRS or BHI and incubated at 37 °C for 24 h. For simulating the gastric condition, 6 mL of a cell suspension were added to the gastric fluid formulation (6.2 g NaCl /L, 2.2 g KCl /L, 0.22 g CaCl_2_ /L, and 1.2 g NaHCO_3_ /L; pH 2.5) with 0.3% pepsin (2500 Units/mg, Sigma-Aldrich) and incubated anaerobically at 37 °C for 1 h with shaking at 150 rpm. After the initial incubation, the resulting viable cell count after 1 h [time 1 (T1)] was determined by plate counts, as described earlier. To simulate small intestinal condition in the next step, 2 mL of the remaining suspension from the gastric simulation were added to 8 mL of intestinal fluid (6.4 g NaHCO_3_ /L, 0.239 KCl g /L, 1.28 g NaCl /L; pH 7.2) with 0.5% bile salts and 0.1% pancreatin, and incubated anaerobically at 37 °C for 3 h with shaking at 150 rpm. The number of viable cells after 3 h [time 2 (T2)] was determined as described earlier. The experiments were performed in triplicate for each of the strains.

Subsequently, the survivability of a mixture of the bacteriocinogenic strain, *E. faecium* ST10Bz, and the test organisms, *E faecalis* 200A or *L. monocytogenes* ATCC^®^15313™, was determined during the GIT simulation. The experiment was done in the same manner as described above, with slight modification. In the simulated gastric condition 3 mL of each strain, *E. faecium* ST10Bz, and either *E. faecalis* 200A or *L. monocytogenes* ATCC^®^15313™, were added to the gastric fluid formulation with 0.3% pepsin and incubated anaerobically at 37 °C for 1 h and at 150 rpm rotation. The same methods were performed in the subsequent steps, as described for the individual strains. After the incubation for cell counting, the plates with distinguishable colonies were counted and pour plated with 1% BHI agar after inoculating with the same test strains, *L. monocytogene*s ATCC^®^15313™ or *E. faecalis* 200A and incubated at 37 °C for 24 h. The colonies observed with inhibition zones were considered as the bacteriocinogenic strain, *E. faecium* ST10Bz, while the colonies with inhibition zones were the test strains. The experiments were performed in duplicates.

#### 2.5.6. Enzyme Profile

The enzymatic activity profile was determined using APIZYM strips (bioMeérieux, Marcy l’Etoile, France) and performed according to the manufacturer’s instructions. The 20 wells (1 control and 19 different substrates) from the APIZYM strips were inoculated with a 24 h-old culture of *E. faecium* ST10Bz grown on MRS agar (BD), and subsequently incubated at 37 °C for 4 h. The evaluation of the activity was recorded using the APIZYM color reaction chart according to the intensity of coloration and the manufacturer’s instructions.

All listed experiments in this study were performed at least in duplicate and on two independent occasions. Standard deviations were calculated. Appropriate statistical analyses were performed.

## 3. Results

### 3.1. Isolation and Molecular Characterization of the Bacteriocinogenic Strain

Application of the triple layer method in the selection of potential bacteriocinogenic strains from *boza* resulted in the selection of a total 71 strains exhibiting inhibitory activity against the applied test strains, *L. monocytogenes* ATCC^®^15313™ and *E. faecalis* 200A (results not shown). However, from all the isolates, only four were found to be of bacteriocinogenic nature. This property of the produced inhibitory substance was confirmed by treating CFS with proteolytic enzymes, and the loss of antimicrobial activity was considered as confirmation of the proteinaceous nature of the antimicrobial substance.

Based on initial characterization, all evaluated isolates were identified as Gram-positive, cocci, and catalase negative presumptive LAB. Differentiation of the four isolates using rep-PCR and RAPD-PCR indicated that the isolates can be considered as replica of the same strain. Consequently, according to the 16S rRNA sequencing, the isolate was identified as *E. faecium* designated the strain number ST10Bz.

The presence of genes related to the production of enterocins A, B, P, and L50B, as well as some other common bacteriocins including nisin and pediocin PA1 (the two commercially available food-grade bacteriocins), were evaluated from the total DNA extracted from *E. faecium* ST10Bz. Initially, enterocins A, B, and P, as well as pediocin PA-1 genes were observed coinciding with their expected sizes. However, when the PCR products were sequenced, the presence of enterocin A and the pediocin PA-1 related gene were invalidated. Based on the analysis of the sequences obtained when targeting enterocin A, only 68.2% similarity was aligned to the enterocin A immunity protein (GenBank: ELA64739.1). The obtained identity is not sufficient to be considered as evidence to the presence of enterocin A, as immunity proteins of different bacteriocin share similar sequences. Moreover, the sequences of the obtained amplicons for enterocins B and P were compared to sequences deposited in the database, as listed in Table 2.

### 3.2. Bacteriocin Production of the Strain

#### 3.2.1. Growth Production

**Figure 1:**
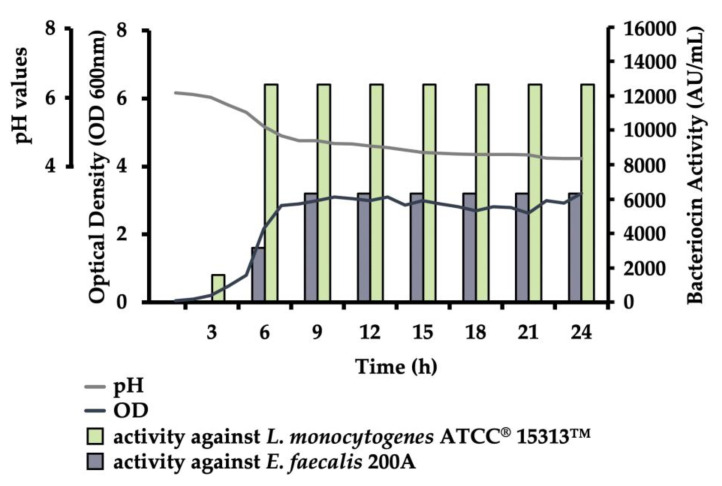
Kinetics of bacterial growth, acidification and bacteriocin production by *E. faecium* ST10Bz. All data represent an average of three repeats. The OD and pH values recorded in each experiment did not differ by more than 5% variation, and standard deviation values were not presented. Identical levels of bacteriocin production (AU/mL) were recorded for all three repeats.

The bacteriocin production of *E. faecium* ST10Bz started during its early exponential growth phase, as early as the 3rd h after inoculation, with 1 600 AU/mL, as shown in Figure 1. It reached the maximum and the stationary phase of bacteriocin production starting on the 9th h of incubation, with 12 800 AU/mL, recorded against *L. monocytogene*s ATCC^®^15313™. The cell density of *E. faecium* ST10Bz increased from 0.048 to 3.21 during the 24-h growth at 37 °C in MRS. The pH, on the other hand, decreased its value from 6.17 to 4.23.

#### 3.2.2. Stability of Bacteriocins

The stability of the bacteriocin produced by *E. faecium* ST10Bz was examined by exposing the antimicrobial to varying temperatures, the presence of common chemicals used in the laboratory and in industry, and to varying pH values. Results showed that none of the evaluated temperatures, chemicals, and pH had any effect on the antimicrobial activity of bacteriocin ST10Bz when evaluated against *L. monocytogenes* ATCC^®^15313™ and *E. faecalis* 200A as indicator strains.

Moreover, after prolonged storage at −20 °C with 12,800 AU/mL initial activity against *L. monocytogenes* ATCC^®^15313™, bacteriocins exhibited the same activity after nine months from initial preparation. A minimal decrease, corresponding to only a two-fold dilution difference from the initial 12,800 AU/mL after ten and eleven months, with 6400 AU/mL bacteriocin activity.

#### 3.2.3. Optimization of Culture Medium

**Figure 2:**
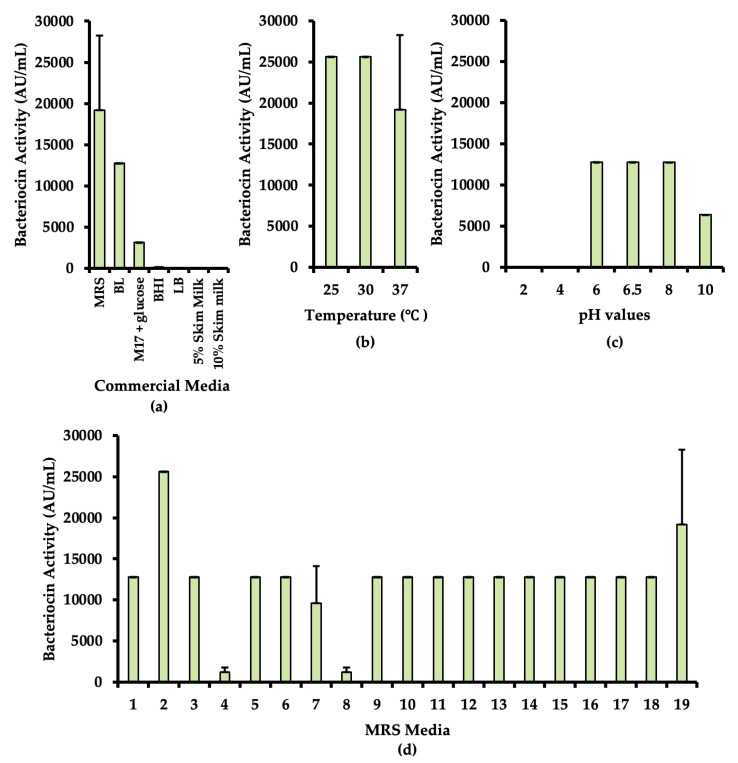
Optimization of medium and environmental conditions for bacteriocin production of *E. faecium* ST10Bz evaluated against *L. monocytogenes* ATCC^®^15313™ as indicator strain. (**a**) With different commercial media, (**b**) at different temperatures, (**c**) different pH-values, and (**d**) different modifications of MRS medium (for information on medium composition see Table S1). In experiments where no differences in levels of bacteriocin production were observed, standard deviation values were not presented.

Different culture media were used to determine the optimal substrate for bacteriocin production. In Figure 2a the highest bacteriocin production was observed in the MRS broth, with 19,200 AU/mL, followed by 12,800 AU/mL in BL broth, 3200 AU/mL in M17 supplemented with 0.5% glucose, and only 200 AU/mL in BHI. Still, in this experiment MRS was considered the best medium for the bacteriocin production of *E. faecium* ST10Bz.

The optimal temperature condition for bacteriocin production was also evaluated. As shown in Figure 2b, elevated bacteriocin production was detected at 25 °C and 30 °C, with 25,600 AU/mL, while at 37 °C only an average of 19 200 AU/mL was observed. Though the yield of bacteriocin was better at low temperatures, a temperature of 37 °C was chosen for the subsequent experiments in order to mimic the normal temperature conditions of the human body, in view of its potential therapeutic application for humans.

When investigating optimal pH conditions for bacteriocin production of *E. faecium* ST10Bz a maximum yield of 12,800 AU/mL was detected when bacteriocin producing strain was cultured MRS broth with pH in the range of 6.0 to 8.0, and with a lower production level at MRS broth with pH 10.0 (6400 AU/mL). Neither growth nor bacteriocin production was observed in MRS broth with pH 2.0 and 4.0.

Finally, the composition of the commercial MRS medium was modified. As shown in Figure 2d, a higher yield of the *E. faecium* ST10Bz bacteriocin was achieved in medium 2 (0.5% glucose; 25 600 AU/mL) and also on medium 19 (2% sucrose; 19 200 AU/mL), as compared with its cultivation in the original MRS medium 3 (2% glucose; 12 800 AU/mL). In comparison with all the sugar modifications, medium 4 (with 5% glucose) sustained the lowest production of bacteriocin (1200 AU/mL).

#### 3.2.4. Bacteriocin Mode of Action

**Figure 3:**
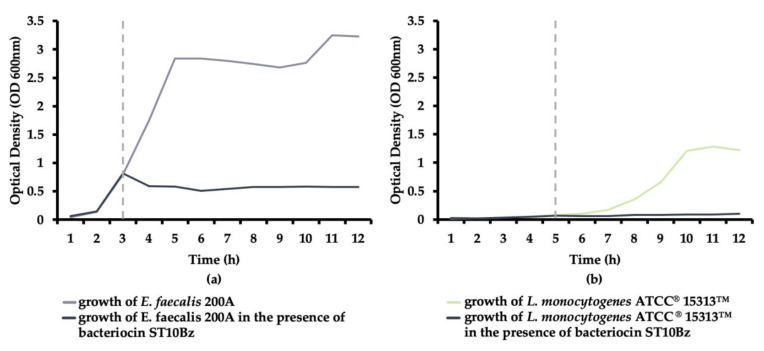
Bactericidal mode of action of the bacteriocin produced by *E. faecium* ST10Bz against (**a**) *E. faecalis* 200A, and (**b**) *L. monocytogenes* ATCC^®^15313™; dotted lines represent the initial exposure to the bacteriocin. All data represent an average of three repeats. The OD values recorded in each experiment did not differ by more than 5% variation and standard deviation values were not presented.

The activity of the bacteriocin produced by *E. faecium* ST10Bz against actively growing *E. faecalis* 200A and *L. monocytogenes* ATCC^®^15313™ was evaluated. None of the test strains showed any growth up until the end of the observation period of 12 h, based on OD readings after addition of the CFS at the 3rd and 5th hour for the *E. faecalis* 200A and *L. monocytogenes* ATCC^®^15313™ cultures, respectively (Figure 3a,b). These results indicate strong inhibitory activity of the bacteriocin and thus, suggest a bactericidal mode of action against the test strains.

#### 3.2.5. Spectrum of Activity

The range of the antimicrobial activity of the *E. faecium* ST10Bz bacteriocin was determined using a total of 127 different strains including several LAB and some pathogens. The CFS from *E. faecium* ST10Bz exhibited inhibitory activity against 41 out of the 44 tested *Enterococcus* strains, including 30 vancomycin-resistant clinical strains, while both *Listeria innocua* strains were inhibited. *Lactobacillus, Leuconostoc, Pediococcus, Weissella, Staphylococcus, Bacillus,* and *Clostridium* strains generally showed higher resistance to the bacteriocin (Table 3).

### 3.3. Safety Tests and Detection of Additional Beneficial Properties

#### 3.3.1. Antibiotic Resistance, Hemolysis, and Biogenic Amine Production

Some microorganisms may develop certain defense mechanisms, allowing their survival even in the presence of antibiotics. The results in Table 1 show that the bacteriocinogenic *E. faecium* ST10Bz was susceptible to all antibiotics tested, except for erythromycin (8 µg/mL), where the MIC observed was two-fold higher than the breakpoint suggested by EFSA. In addition to the antibiotic susceptibility test, the presence of factors related to vancomycin resistance genes, van A, B, C, D, E, and G, was evaluated from the total DNA extracted from *E. faecium* ST10Bz. Initially, the *van*A gene was observed, coinciding with the expected size of the amplicon. However, when the PCR product was sequenced, presence of the *van*A gene was invalidated. On the other hand, hemolysis safety test results showed that *E. faecium* ST10Bz was either gamma- or non-hemolytic. Finally, *E. faecium* ST10Bz showed capability of producing only tyramine among the biogenic amines tested for (data not shown).

Antibiotic resistance in beneficial microorganisms is a delicate issue. On the one hand, possible spread via horizontal gene transfer of antibiotic resistance genes from beneficial to other inhabitants of GIT (other beneficial or pathogenic microorganisms) can result in serious health issues. But on the other hand, the presence of limited antibiotic resistance in beneficial strains can be considered as acceptable feature, as the organism may be applied in combination with a specific antibiotic and exhibit synergistic effect, which can be explored as potential alternative treatments of diseases in human and veterinary medical practices.

#### 3.3.2. Detection of Adhesion and Gamma Aminobutyric Acid (GABA) Production Related Genes

Adhesion genes are important factors facilitating the adhesion of a probiotic strain to the intestinal mucosal layer and thereby supporting its colonization in the GIT. Based on the analysis of the generated amplicons, the *map*A, *mub*, and *ef*-Tu genes were detected for *E. faecium* ST10Bz, yet *prg*B, *EF*1249, *EF*2380, and *EF*2662 were not found. In addition, the performed PCR targeting of the *gad* gene generated positive results for potential GABA production by *E. faecium* ST10Bz.

#### 3.3.3. GIT Model

**Figure 4:**
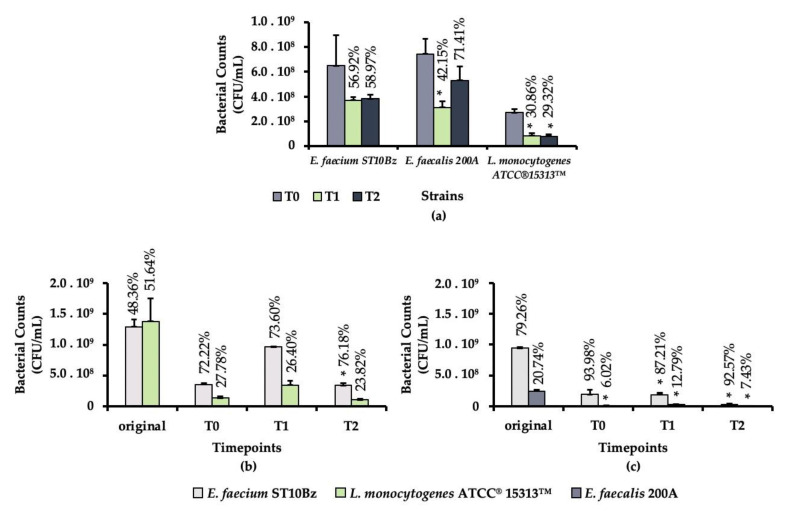
(**a**) Survivability of the individual strains under gastrointestinal tract (GIT) conditions. Proportional presence of *E. faecium* ST10Bz and test organisms: (**b**) co-cultures of *E. faecium* ST1 and *L. monocytogenes* ATCC^®^15313™, (**c**) co-cultures of *E. faecium* ST10Bz and *E. faecalis* 200A. Timepoints T1 and T2 represent exposure times of either 1 h or 3 h simulation of the stomach or small intestine, respectively. Asterisks indicate statistically significant differences.

In vitro simulation of the GIT was used to evaluate the ability of the *E. faecium* ST10Bz to withstand the stressful conditions of the gastrointestinal environment. The bacteriocinogenic strain *E. faecium* ST10Bz and the test strains, *L. monocytogenes* ATCC^®^15313™ and *E. faecalis* 200A, were subjected to GIT stress, both individually and in mixed cultures. Under individual exposure to the simulated GIT conditions, *E. faecium* ST10Bz and *E. faecalis* 200A showed higher survivability after 3 h (T2) under the small-intestinal simulation, as compared to *L. monocytogenes* ATCC^®^15313™. Co-cultures of *E. faecium* ST10Bz with either *L. monocytogenes* ATCC^®^15313™ (Figure 4b) or *E. faecalis* 200A (Figure 4c) showed higher survivability of *E. faecium* ST10Bz than that of the individual test strains. As compared to the percentage change in survival of the individual strains to the same strains in the mixed culture experiments, the test strains showed higher reduction rates. Therefore, the test strains were notably reduced in the presence of the bacteriocinogenic strain *E. faecium* ST10Bz.

#### 3.3.4. Enzymes Production Profile

Using the APIZYM test enzymatic activities of *E. faecium* ST10Bz could be detected for esterase (C4), esterase lipase (C8), leucine arylamidase, valine arylamidase, cysteine arylamidase, α-chymotrypsin, acid phosphatase, naphthol-AS-BI-phosphohydrolase and β-galactosidase.

## 4. Discussion

Over the years, *boza* has been intensively studied for its claimed benefits to consumer health, primarily based on its prebiotic and probiotic features [39]. Numerous bacteriocinogenic LAB strains were previously isolated from the beverage, including *Lb. plantarum* strains ST19BZ, ST414BZ, and ST664BZ, *Lb. pentosus* ST712BZ, *Lb. rhamnosus* ST461BZ and ST462BZ, and *Lb. paracasei* ST242BZ and ST284BZ [40], *Leuconostoc pseudomesenteroides* KM432Bz [41], *Lb. plantarum* JW3BZ and JW6BBZ, *Lb. fermentum* JW11BZ and JW15BZ [42], and *E. faecium* YT52 [43]. In this study, the isolation of bacteriocinogenic *E. faecium* from *boza* can be considered as a further contribution towards a better characterization of its microbial consortium and its beneficial properties.

Bacteriocins, by definition, are ribosomally-synthesized antimicrobial peptides [10], while proteins and peptides are degraded by proteolytic enzymes into smaller units. Therefore, sensitivity of antimicrobial metabolite/s from *E. faecium* ST10Bz to proteolytic enzymes can serve as a key point in the characterization and differentiation (from other antimicrobial metabolites) of the extracted compound(s), thereby pointing to its proteinaceous nature [44].

Bacteriocin production is dependent on expression of the appropriate genes. Some bacteriocinogenic strains carry genetic determinants for more than one bacteriocin; however, expression of these genes simultaneously or individually depends either on environmental conditions or on induction by specific factors. This characteristic has been observed and described previously for strains of *Leuconostoc pseudomesenteroides* QU 15, producing leucocins A-QU 15, Q, and N [45], for *Lb. gasseri* LM19, expressing three bacteriocins, including a novel bacteriocin, gassericin M [46], *Lactococcus lactis* LMG2081 with lactococcin and lacticin LMG [47], and *E. faecium* NKR-5-3 with enterocins NKR-5-3A, B, C, D, and Z [48]. In this study, genes for the production of enterocins B and P were detected for *E. faecium* ST10Bz. However, further purification and identification must be performed to finally verify the identity of these bacteriocins.

The production of bacteriocins was observed starting with the early exponential growth phase of the strain. The ability to produce the bacteriocin at the aforementioned time point indicates that the bacteriocin could be considered as a primary metabolite of this strain [49]. Similar kinetics were previously observed for the bacteriocinogenic enterococci *E. faecalis* AP45 [50], *E. faecium* B3L3 [51], *E. faecium* RZS C5 [52], and *E. faecium* ST311LD [53].

In this study, the stability of the bacteriocin(s) to varying environmental conditions was also checked in order to evaluate its potential as antimicrobial in the food industry and in research laboratory settings. The bacteriocin produced by *E. faecium* ST10Bz was tested for its stability to varying temperatures that may possibly be encountered in the food industry, as well as in laboratory settings, ranging from 5.4 °C–121 °C, representing storage, production and sterilization processes [54]. The molecular size of bacteriocins is generally smaller than 10 kDa [7], thus, relative to their structure these compounds are stable during thermal treatment. This may render them suitable for application in the production of different fermented food products where parts of the technological processes include rapid (pre-)heating to high temperatures, and considering laboratory sterilization processes at 121 °C for 15 min.

During the food production process and in the laboratory, food or laboratory samples are commonly exposed to different chemicals. Thus, it is important for an antimicrobial compound to retain its activity in the presence of specific contaminants and different chemicals. This characteristic was also reported for other enterocins, including EJ97 [55], bozacin B14 [56], and enterocins ST209GB, ST278GB, ST315GB, and ST711GB [26]. This implies the potential of such bacteriocins to be used together with common chemicals and still act as antimicrobials in the bio-preservation processes. This feature will also facilitate the planning and performing of different purification and identification procedures for the antimicrobial peptides.

Moreover, pH is an important technological characteristic, especially for fermented products with low acidity levels. The bacteriocin produced by *E. faecium* ST10Bz remained stable when exposed to different pH-values and maintained its activity against the test strains used in this study (Table 3). These results are in agreement with previous reports for other bacteriocins by Urso et al. [57], Todorov et al. [25] and Favaro et al. [26].

The stability of the bacteriocin at a storage temperature of −20 °C was evaluated over time to determine its shelf life. It was previously reported that some bacteriocins, especially those of *Lb. acidophilus* DSM 20079 [58] and *Lb. plantarum* C19 [59], retained their activity after 90 and 180 days of storage at −20 °C, respectively. In contrast, others like the bacteriocin from *P. acidilactici* kp10 could be affected by prolonged storage conditions, losing their inhibitory activity against *L. monocytogenes, Escherichia coli,* and *Staphylococcus aureus* immediately after three and six months of storage at −20 °C, with 87% reduction [60]. Therefore, the bacteriocin/s produced by *E. faecium* ST10Bz in this study showed good retention of activity and could retain a stable inhibitory property even during prolonged storage.

In the determination of optimal conditions for the bacteriocin production of *E. faecium* ST10Bz, MRS was found to be the optimal medium, thus, the subsequent experiments were conducted using the same medium with some alterations in the temperature, pH, and composition of the medium. Previous studies showed that bacteriocin production is often dependent of or correlated with cell growth, and therefore the best bacteriocin yield would be expected at the optimal growth temperature of a strain [61], as was also observed for the production of plantaricin produced by *Lb. plantarum* ZJ316 [62], and enterocin 1146 [63]. In addition, *Enterococcus* spp. are well known for their acid tolerance and ability to grow both at high and low pH levels ranging from pH 4.5–10.0 [64]. Such characteristic of bacteriocinogenic *E. faecium* ST10Bz can be considered as a positive feature and may play an important role in the application of the strain as probiotic or starter culture in food fermentation processes. Moreover, humans and animals interact with different ecological environments with varying pH values, most specifically in the GIT. The ability of such potential probiotics to grow and produce bacteriocins over a wide pH range may thus support their probiotic potential and render them promising candidates for future application. Lastly, with alteration of the composition of the medium, the strain gave higher yield of bacteriocin in the medium with lower glucose concentration; this was also observed for *E. faecium* DPC1156 [65]. This phenomenon can be explained by substrate inhibition where a high substrate concentration decreases the rate of growth and bacteriocin production of the organism [65,66]. On the other hand, high bacteriocin production was also observed in MRS medium with 2% sucrose. The same activity was reported for nisin production by the strain *Lc. lactis* subsp. *lactis* NIZO 22186 [67]. According to Abbasiliasi et al. [68], the ability of a strain to produce higher bacteriocin levels in a medium when exchanging glucose with other sugars such as sucrose may be explained by the presence of a complex enzymatic system that could support the utilization of complex sugars. Therefore, a higher bacteriocin production with a low-priced complex sugar as carbon source may enable a cost-effective bacteriocin production.

In the evaluation of the mode of action of *E. faecium* ST10Bz against the two test strains, the retention of the OD level of the cultures after addition of the CFS, up to the 12th h, indicated a strong inhibitory activity of the bacteriocin, and thus suggests a bactericidal mode of action against the test strains. Similar observations were found for different bacteriocinogenic strains of *E. faecium* from Bulgarian feta cheese [26].

Adsorption or attachment of the bacteriocin to the test strain initiates the activity of the antimicrobial. From previous studies, *E. mundtii* CRL35 showed optimal adsorption to *L. monocytogenes* at pH 4.0 and temperatures ranging from 20–37 °C, with decreasing adsorption as the pH increases; for *E. faecium* ST88Ch the optimal adsorption conditions were at 37 °C and pH 4.0, and at 30–37 °C at pH 6.0 [69]. These results showed that the adsorption of bacteriocins vary with different environmental conditions. Moreover, all results can be used in the prediction of efficacy of a bacteriocin for possible application in food bio-preservation processes.

For the spectrum of activity of the bacteriocin(s) produced by *E. faecium* ST10Bz, the results support the hypothesis that the antimicrobials present in the CFS were bacteriocins with inhibitory activity against closely related species [10]. Specific activity against important pathogens such as *L. monocytogenes* and vancomycin resistant *Enterococcus* strains is an important characteristic of the bacteriocin produced by *E. faecium* ST10Bz. It is also of interest that most of the tested *Lactobacillus* strains were not affected by the bacteriocin; these strains included a well-known probiotic strain, *Lb. rhamnosus* LGG^®^, and other safe in-house isolates of the HEM culture collection. This characteristic suggests the potential of this bacteriocin for controlling listerial and enterococcal infections in combination with some probiotic strains.

Horizontal gene transfer of genetic material between bacteria has been identified as a major factor contributing to an increasing health risk posed by antibiotic resistance; this may serve to worsen the potential threat when pathogenic bacteria carry virulence factors [70]. *Enterococcus* strains were generally considered as “prepared” for the antibiotic era, as they usually carry resistance genes intrinsic to their genetic make-up, reducing their sensitivity to a wide range of antibiotics and biocides [71,72]. The emergence of a specific group of (mainly nosocomial) enterococcal strains known as vancomycin-resistant enterococci (VRE), currently constitutes a serious health problem in medical (human and veterinary) practices [73].

Hemolysis, or the ability of bacteria to lyse red blood cells, strongly contributes to the virulence of potentially pathogenic strains against humans and animals [74]. On the other hand, biogenic amines (BAs) are primarily low-molecular weight organic compounds that may be formed by microbial decarboxylation of some amino acids in foods. Although supporting diverse physiological functions, deleterious effects such as nausea and headaches may be experienced at higher concentrations, especially by sensitive consumers. Histamine and tyramine are those BAs most frequently associated with intoxications such as “scombroid fish poisoning” and “cheese reaction”, respectively [75]. *E. faecium* ST10Bz shows the ability to produce tyramine, which, however, is a common physiological characteristic of the genus *Enterococcus* [76]. Its ability to produce tyramine might render it unsafe for people using monoamine oxidase inhibitor (MAOI) due to its interaction with tyramine that could cause a potentially fatal hypertensive crisis. The hypertensive crisis was described as having sudden but severe, pulsating headache, palpitations, diaphoresis, stiff neck, and nausea, which could also possibly lead to stroke [77]. On the other hand, the dynamics of tyramine production by (putative) probiotic enterococci has not been investigated yet. Further clarification of this issue is essential in view of potential BA production by some probiotic strains, and open questions regarding decarboxylation activity under (simulated) GIT conditions.

Adhesion genes are important in the production and expression of proteins mediating bacterial adherence to the intestinal epithelium. These functional factors support the colonization of the GIT, which can be considered as beneficial characteristics of the newly isolated strain in support of its evaluation as a probiotic. However, these genes do not always express as a positive feature that would contribute to the probiotic potential of the investigated LAB strain. Additional tests should be performed in order to determine whether these genes are fully expressed under environmental conditions specific to the human and animal GIT. Even if strains have successful adhesion to the GIT, additional beneficial properties need to be expressed by probiotic candidates, as part of additional experiments and evaluation. In this study, only the genes *map*A, *mub,* and *EF*-Tu were detected, similar to that of *E. faecium* ST311LD [36]. Adhesion genes in a probiotic candidate strain may be regarded as a positive characteristic, especially for strains producing antimicrobials. Their ability to adhere to other strains may allow easier inhibitory activity of deleterious organisms. From another angle, this feature may be considered negative primarily due to the possible initiation of gene transfer. Therefore, further investigations need to be conducted to prove the safety of this strain.

In vitro simulation of the GIT was used to evaluate the ability of the *E. faecium* ST10Bz to withstand the stressful conditions of the gastrointestinal environment. The ability of the bacteriocinogenic strain to survive GIT conditions indicates its potential as probiotic and suggests beneficial properties such as its survival, growth, and performance in this ecosystem. Generally, *Enterococcus* spp. are capable of surviving even after drastic changes of the environmental conditions in the GIT, varying from acidic to basic, and the subsequent presence of bile salts [78]. In addition, the survivability of *E. faecium* ST10Bz, when mixed with the test strains, was higher. Thus, in general, *E. faecium* ST10Bz may possibly attenuate listerial and enterococcal infections under conditions of the upper GIT. Moreover, *E. faecium* ST10Bz was able to significantly reduce the levels of *L. monocytogenes* ATCC^®^15313™ and *E. faecalis* 200A; this may be considered as an example of superior competitiveness [79]. It could be speculated that under conditions mimicking GIT conditions bacteriocin/s are most probably produced and are able to inhibit the applied test organisms. An appropriate animal model should be used to prove this hypothesis.

Production of specific enzymes plays an important role not only in the normal metabolism of bacterial species, but also by potentially amplifying probiotic benefits of a specific LAB strain. Previously Park et al. [80] reported that different strains of *Bacillus* spp. isolated from Korean traditional fermented food products may have different expression levels of proteolytic enzymes, and, related to these specific characteristics, some strains have shown potential for application as anti-obesity probiotics. In industrial and biotechnological applications of beneficial microbial cultures, the ability to degrade amino acids into volatile molecules is related to the organoleptic complexity of fermented meat products. Montel et al. [81] showed that the aldehydes, alcohols, and acids, derived from the breakdown of leucine, valine, phenylalanine, and methionine, have minimal effects on the sensory quality, taste, and smell of the fermented products. Presence of β-galactosidase is considered as a beneficial property of LAB strains applied as probiotics, since this enzyme is directly involved in the breakdown of lactose and is considered as an essential for probiotics intended for lactose intolerant individuals [19]. Additionally, the glutamate decarboxylase (*gad*) gene encoding the enzyme responsible for the conversion of glutamate to GABA [82], was found in the genome of strain ST10Bz. GABA production is considered a beneficial property for a probiotic strain. According to Bajić et al. [38], GABA plays a protective role in different diseases, such as type 1 diabetes [83], autoimmune encephalomyelitis [84], arthritis [85,86], and contact dermatitis [87]. Therefore, the presence of the *gad* gene can be considered as an additional beneficial property of a putative probiotic strain.

## 5. Conclusions

Bacteriocin produced by *E. faecium* ST10Bz possesses a strong anti-listerial and anti-enterococcal activity, thus rendering the antimicrobial metabolite a potential therapeutic alternative to antibiotics for application against these opportunistic pathogens. Beneficial properties and safety of *E. faecium* ST10Bz were evaluated. These results suggest that *E. faecium* ST10Bz can be considered as a promising potential probiotic strain with a biotherapeutic potential.

## Figures and Tables

**Table 1:** Minimum inhibitory concentration of different antibiotics experimentally determined for *Enterococcus faecium* ST10Bz and *Enterococcus faecalis* ATCC^®^29212™ compared with the respective breakpoints proposed by European Food Safety Authority EFSA and Clinical and Laboratory Standards Institute (CLSI).

	µg/mL									
	Ampicillin	Vancomycin	Gentamicin	Kanamycin	Streptomycin	Erythromycin	Clindamycin	Tylosin	Tetracycline	Chloramphenicol
Breakpoints for *E. faecium* according to EFSA	2	4	32	1024	128	4	4	4	4	16
Experimental values for *E. faecium* ST10Bz	0.5	0.5	32	128	32	8	0.5	1	0.5	8
Quality control strain range for *E. faecalis* ATCC^®^29212^TM^ according CLSI	0.5–2	1–4	4–16	16–64	−	1–4	4–16	-	8–32	4–16
Experimental values for *E. faecalis* ATCC^®^29212^TM^	1	2	8	16	32	2	16	1	16	8

**Table 2:** The putative protein sequences of enterocins B and P generated from the nucleotide sequence of the amplicons; results of the PCR targeting appropriate bacteriocin genes in the genome of *E. faecium* ST10Bz in comparison to that of known enterocins B and P. Differences in amino acids are indicated in bold and underlined.

	Sequences
Enterocin B	
*E. faecium* ST10BZ	**CHINLFP**T**PVITFVCCF**EN DHRMPNELNR PNNLSKGGAK CGAAIAGGLF GIPKGPLAWA AGLANVYS
GenBank: AAD28234.1	**MQNVKELS**T**KEMKQIIGG**EN DHRMPNELNR PNNLSKGGAK CGAAIAGGLF GIPKGPLAWA AGLANVYS**KC****N**
Enterocin P	
*E. faecium* ST10BZ	MRKKLFSLTL IGKFGLVVTN FGTKVDAATR SYDNGIYCNN SKCWVNWGEA KENIAGIVIS GWASGLAGM
GenBank: ACU28817.1	MRKKLFSLTL IGKFGLVVTN FGTKVDAATR SYDNGIYCNN SKCWVNWGEA KENIAGIVIS GWASGLAGM

**Table 3:** Spectrum of activity of bacteriocin produced by *E. faecium* ST10Bz showing the number of sensitive strains out of the total tested for a specific species.

Species	Total
*Enterococcus avium*	1/3
*Enterococcus faecium*	6/6
*Enterococcus faecalis*	2/2
*Enterococcus gallinarum*	0/1
*Enterococcus thailandicus*	1/1
*Enterococcus durans*	1/1
Vacomycin-resistant *Enterococcus* strains (VRE)	30/30
*Listeria monocytogenes*	1/1
*Listeria innocua*	2/2
*Bacillus cereus*	0/2
*Bacillus subtilis*	0/1
*Bacillus licheniformis*	0/1
*Bacillus pumilus*	0/1
*Clostridium butyricum*	0/1
*Pediococcus acidilactici*	1/1
*Pediococcus pentosaceus*	1/4
*Weissella cibaria*	0/1
*Leuconostoc mesenteroides*	0/6
*Staphylococcus aureus*	0/1
*Lactobacillus rhamnosus*	0/5
*Lactobacillus paracasei*	0/5
*Lactobacillus plantarum*	0/9
*Lactobacillus sanikiri*	0/4
*Lactobacillus brevis*	0/3
*Lactobacillus salivarius*	0/3
*Lactobacillus sakei*	1/4
*Lactobacillus curvatus*	0/2
*Lactobacillus fermentum*	0/2
*Staphylococcus epidermidis*	0/1
*Staphylococcus cohnii* subsp. *cohnii*	0/1
*Staphylococcus warneri*	0/1
*S* *taphylococcus simulans*	0/1
*Staphylococcus capitis* subsp. *capitis*	0/1
*S* *taphylococcus lentus*	0/1
*Staphylococcus carnosus* subsp. *carnosus*	0/1
*S* *taphylococcus auricularis*	0/1
*S* *taphylococcus arlettae*	0/1
*S* *thaphylococcus delphini*	0/1
*Streptococcus sanguinis*	0/1
*Streptococcus mitis*	0/1
*Streptococcus gordonii*	0/1
*Streptococcus mutans*	0/1

## Supplementary Materials

The following are available online at https://www.mdpi.com/2076-2607/8/10/1474/s1, Table S1: Composition of the media in evaluation of the effect of MRS medium ingredients on the production of bacteriocin by *Enterococcus faecium* ST10Bz, Table S2: Spectrum of activity of bacteriocin produced by *Enterococcus faecium* ST10Bz.
