# Supplementary material for: Exploring Beneficial Properties of the Bacteriocinogenic Enterococcus faecium ST10Bz Strain Isolated from Boza, a Bulgarian Cereal-Based Beverage
Source: Microorganisms. 2020 Sep 25;8(10):1474. doi: 10.3390/microorganisms8101474 (PMC7600690; doi:10.3390/microorganisms8101474)
Supplement: Supplementary file 1 [file microorganisms-08-01474-s001.pdf]

Supplementary Table S1. Composition of the media assessed for evaluating and comparing the effect of conventional MRS medium on the production of bacteriocin by *Enterococcus faecium* ST10Bz.

**Components**	**g/L**																		
	**1**	**2**	**3**	**4**	**5**	**6**	**7**	**8**	**9**	**10**	**11**	**12**	**13**	**14**	**15**	**16**	**17**	**18**	**19**
peptone	10	10	10	10	10	0	10	10	10	10	10	10	10	10	10	10	10	10	10
beef extract	10	10	10	10	10	10	0	10	10	10	10	10	10	10	10	10	10	10	10
yeast extract	5	5	5	5	0	5	5	5	5	5	5	5	5	5	5	5	5	5	5
sugar	10	5	20	50	20	20	20	20	20	20	20	20	20	20	20*	20*	20*	20*	20*
tween 80	1	1	1	1	1	1	1	0	1	1	1	1	1	1	1	1	1	1	1
ammonium citrate	2	2	2	2	2	2	2	2	2	2	2	2	2	2	2	2	2	2	2
sodium acetate	5	5	5	5	5	5	5	5	5	5	5	5	5	5	5	5	5	5	5
magnesium sulfate	0.1	0.1	0.1	0.1	0.1	0.1	0.1	0.1	0.1	0.1	0	0.2	0.1	0.1	0.1	0.1	0.1	0.1	0.1
manganese sulfate	0.05	0.05	0.05	0.05	0.05	0.05	0.05	0.05	0	0.1	0.05	0.05	0.05	0.05	0.05	0.05	0.05	0.05	0.05
dipotassium phosphate	2	2	2	2	2	2	2	2	2	2	2	2	0	4	2	2	2	2	2

*15 – lactose; 16 – maltose; 17 – trehalose; 18 – fructose; 19 - sucrose.

Supplementary Table S2. Spectrum of activity of the bacteriocin produced by *Enterococcus faecium* ST10Bz. BHI: Brain-Heart Infusion; RCM: Reinforced Clostridial Medium; MRS: de Man, Rogosa, Sharpe.

Species	Code	Origin	Growth conditions
Enterococcus avium	NGP 20		BHI ; 37°C; aerobic
	NGP 21		BHI ; 37°C; aerobic
	1051	human baby feces	BHI ; 37°C; aerobic
Enterococcus faecium	264	human baby feces	BHI ; 37°C; aerobic
	888	Mexican sheep cheese	BHI ; 37°C; aerobic
	1108	tomato	BHI ; 37°C; aerobic
	8	commercial cow milk	BHI ; 37°C; aerobic
	59	Mexican cheese	BHI ; 37°C; aerobic
	222	kimchi	BHI ; 37°C; aerobic
Enterococcus faecalis	688		BHI ; 37°C; aerobic
	263	baby feces	BHI ; 37°C; aerobic
Enterococcus gallinarum	107	Mexicana curd	BHI ; 37°C; aerobic
Enterococcus thailandicus	237	doenjang	BHI ; 37°C; aerobic
Enterococcus durans	761	human adult feces	BHI ; 37°C; aerobic
Vancomycin-resistant	2	clinical isolates	BHI ; 37°C; aerobic
Enterococcus strains (VRE)	6	clinical isolates	BHI ; 37°C; aerobic
	7	clinical isolates	BHI ; 37°C; aerobic
	8	clinical isolates	BHI ; 37°C; aerobic
	11	clinical isolates	BHI ; 37°C; aerobic
	13	clinical isolates	BHI ; 37°C; aerobic
	15	clinical isolates	BHI ; 37°C; aerobic
	16	clinical isolates	BHI ; 37°C; aerobic
	18	clinical isolates	BHI ; 37°C; aerobic
	19	clinical isolates	BHI ; 37°C; aerobic
	21	clinical isolates	BHI ; 37°C; aerobic
	22	clinical isolates	BHI ; 37°C; aerobic
	23	clinical isolates	BHI ; 37°C; aerobic
	25	clinical isolates	BHI ; 37°C; aerobic
	27	clinical isolates	BHI ; 37°C; aerobic
	29	clinical isolates	BHI ; 37°C; aerobic
	30	clinical isolates	BHI ; 37°C; aerobic
	33	clinical isolates	BHI ; 37°C; aerobic
	34	clinical isolates	BHI ; 37°C; aerobic
	35	clinical isolates	BHI ; 37°C; aerobic
	36	clinical isolates	BHI ; 37°C; aerobic
	37	clinical isolates	BHI ; 37°C; aerobic
	38	clinical isolates	BHI ; 37°C; aerobic

	39	clinical isolates	BHI ; 37°C; aerobic
	40	clinical isolates	BHI ; 37°C; aerobic
	43	clinical isolates	BHI ; 37°C; aerobic
	45	clinical isolates	BHI ; 37°C; aerobic
	46	clinical isolates	BHI ; 37°C; aerobic
	47	clinical isolates	BHI ; 37°C; aerobic
	48	clinical isolates	BHI ; 37°C; aerobic
Leuconostoc mesenteroides	509		BHI ; 37°C; aerobic
	445	ant	BHI ; 37°C; aerobic
	109	Mexican curd	BHI ; 37°C; aerobic
	695	human adult feces	BHI ; 37°C; aerobic
	357	white kimchi	BHI ; 37°C; aerobic
	104	Mexican cream	BHI ; 37°C; aerobic
Weissella cibaria	1029		BHI ; 37°C; aerobic
Staphylococcus aureus	ATCC 6538		BHI ; 37°C; aerobic
Pediococcus pentosaceus	223	kimchi	BHI ; 37°C; aerobic
	785	fermented oyster	BHI ; 37°C; aerobic
	805	New Zealand cheese	BHI ; 37°C; aerobic
	259	kimchi	BHI ; 37°C; aerobic
Pediococcus acidilactici	867	human baby feces	BHI ; 37°C; aerobic
Listeria monocytogenes	ATCC 15313		BHI ; 37°C; aerobic
Listeria innocua	ATCC 33090		BHI ; 37°C; aerobic
	KCTC 3586		BHI ; 37°C; aerobic
Bacillus cereus	ATCC 27348		BHI ; 30°C; aerobic
	ATCC 11778		BHI ; 30°C; aerobic
Bacillus subtilis	KCTC 3135		BHI ; 30°C; aerobic
Bacillus pumilus	KCTC 3348		BHI ; 30°C; aerobic
Bacillus licheniformis	KCTC 1918		BHI ; 30°C; aerobic
Clostridium butyricum	ATCC 19398		RCM; 30°C; anaerobic
Lactobacillus rhamnosus	42	feces	MRS ; 37°C; aerobic
	647	peach	MRS ; 37°C; aerobic
	897	human baby feces	MRS ; 37°C; aerobic
	911	human baby feces	MRS ; 37°C; aerobic
	LGG		MRS ; 37°C; aerobic
Lactobacillus paracasei	532	kimchi	MRS ; 37°C; aerobic
	973	human baby feces	MRS ; 37°C; aerobic
	987	human baby feces	MRS ; 37°C; aerobic
	848	human baby feces	MRS ; 37°C; aerobic
	268	human baby feces	MRS ; 37°C; aerobic
Lactobacillus brevis	384	kimchi	MRS ; 37°C; aerobic

	803	sesame leaves	MRS ; 37°C; aerobic
	896	Mexican sheep cheese	MRS ; 37°C; aerobic
Lactobacillus curvatus	499	Mexican cheese	MRS ; 37°C; aerobic
	643	Mexican cheese	MRS ; 37°C; aerobic
Lactobacillus fermentum	792	fermented rice	MRS ; 37°C; aerobic
	899	human baby feces	MRS ; 37°C; aerobic
Lactobacillus salivarius	851	human saliva	MRS ; 37°C; aerobic
	852	human saliva	MRS ; 37°C; aerobic
	1043	human baby feces	MRS ; 37°C; aerobic
Lactobacillus sakei	253	kimchi	MRS ; 37°C; aerobic
	686	human adult feces	MRS ; 37°C; aerobic
	802	sesame leaves	MRS ; 37°C; aerobic
	1074	avocado	MRS ; 37°C; aerobic
Lactobacillus plantarum	30	kimchi	MRS ; 37°C; aerobic
	187	peach	MRS ; 37°C; aerobic
	211	fish doenjang and gochujang	MRS ; 37°C; aerobic
	388	cheese	MRS ; 37°C; aerobic
	635	human baby feces	MRS ; 37°C; aerobic
	641	dog feces	MRS ; 37°C; aerobic
	660	human baby feces	MRS ; 37°C; aerobic
	201	seaweed gochujang	MRS ; 37°C; aerobic
	340	insect	MRS ; 37°C; aerobic
Lactobacillus sanikiri	742	goat feta cheese	MRS ; 37°C; aerobic
	757	fermented milk (Bulgaros)	MRS ; 37°C; aerobic
	764	human adult feces	MRS ; 37°C; aerobic
	772	buffalo cheese	MRS ; 37°C; aerobic
Staphylococcus epidermidis	KACC 13234		BHI ; 37°C; aerobic
Staphylococcus cohnii subsp. *cohnii*	KACC 13237		BHI ; 37°C; aerobic
Staphylococcus warneri	KACC 13240		BHI ; 37°C; aerobic
Staphylococcus simulans	KACC 13241		BHI ; 37°C; aerobic
Staphylococcus capitis subsp. *capitis*	KACC 13242		BHI ; 37°C; aerobic
Staphylococcus lentus	KACC 13245		BHI ; 37°C; aerobic
Staphylococcus carnosus subsp. *carnosus*	KACC 13250		BHI ; 37°C; aerobic
Staphylococcus auricularis	KACC 13252		BHI ; 37°C; aerobic
Staphylococcus arlettae	KACC 13254		BHI ; 37°C; aerobic
Staphylococcus delphini	KACC 13258		BHI ; 37°C; aerobic

Streptococcus sanguinis	KACC 11301	BHI ; 37°C; aerobic
Streptococcus mitis	KACC 16832	BHI ; 37°C; aerobic
Streptococcus gordonii	KACC 13829	BHI ; 37°C; aerobic
Streptococcus mutans	KACC 16833	BHI ; 37°C; aerobic
